# Correction of the Size-of-Source Effect in Thermal Imaging and Application to Body Thermometry

**DOI:** 10.3390/s26072177

**Published:** 2026-03-31

**Authors:** Erik Bryan Beall

**Affiliations:** ECB Technologies LLC, Davidson, NC 28036, USA; ebeall@gmail.com; Tel.: +1-440-941-5489

**Keywords:** size-of-source effect, body thermometry, long-wavelength infrared, febrile, thermographic screening, microbolometer, artifact correction, medical thermometry

## Abstract

Single-element bolometers have been widely used for medical thermometry to predict a core body temperature based on the measured surface temperature and a pre-determined clinical correlation. The size-of-source effect (SSE) in bolometers is recognized as an important source of error and has been extensively studied such that SSE can be controlled sufficiently to obtain the required accuracy. Thermal imaging cameras relying on much of the same principles have also been widely used for medical thermometry, but recent work has shown that SSE in thermal imaging differs from SSE in single-element bolometers. An unappreciated aspect of this artifact in thermal imaging has been its outsize impact on accuracy, producing more than a degree of inaccuracy in typical scenarios. However, because the artifact has so far avoided a satisfactory characterization, this impact has not been incorporated into the standards and expert recommendations for body thermometry with thermal imaging. In this work, we characterize SSE-like artifact as proportional to the difference between source and non-source temperatures as a function of source size, showing that the same percent deviation artifact is obtained at different source temperatures. We then derive an objective method to obtain convolution kernel parameters and apply these to several thermal imaging sensors and optics configurations. Finally, we show that these methods are sufficient to achieve the required accuracy for body thermometry with thermal imaging.

## 1. Introduction

The measurement accuracy of radiation thermometry has long been known to depend on the size of the object being measured within the sensor’s optical field of view (FOV). This dependence was termed the size-of-source effect (SSE) and was initially understood to be a function of the geometric proportion of the FOV filled by a source of interest having a uniform temperature and being a function of optical effects from imperfections, classical scattering and Fraunhofer diffraction [1]. The direct method to empirically determine a system’s SSE measures the reduction versus target size by using a fixed target or aperture size and varying distance to target or alternatively a varying aperture occluding the target [2]. The indirect method measures the reduction using a fixed occluding disk at or near the nominal target size in the center of the FOV over a calibration source having an aperture larger than the occluding target while varying the size of the aperture [3]. Scanning methods enable the automation of SSE characterization [4,5] and various analytical forms have been validated for both methods across a wide range of pyrometers with measurements made at several national laboratories [6]. For modern devices, manufacturers typically specify a nominal target size at one distance or a distance-to-spot ratio [7,8]. Proper specification is generally accepted as a critical component of the solution to the SSE problem in single-element radiation thermometers. We hereafter refer to the currently-understood single-element SSE as SSSE.

For multi-element long-wavelength infrared (LWIR) sensor arrays, the FOV of a single-element bolometer is analogous to the instantaneous FOV (iFOV) of a multi-element array imaging bolometer. The iFOV is then the FOV of the focused scene covering the extent of the array divided by the array length in one axis, although the analogy deviates more for non-central pixels, and especially for arrays having wider optical FOVs. Continuing the analogy, if the object is not perfectly centered on or is not fully subtended by a pixel, the measurement at the pixel will be affected by subsampling. To avoid this subsampling, it is generally recommended that the object must fill at least 3 times the iFOV (in two dimensions, its illumination must be able to fill a 3 × 3 grid of pixels) to ensure the radiation emitted by an object is fully subtended by at least one pixel, but please note this overly simplistic view is made here solely to point out the partial illumination effect that will be present at the boundaries of objects. The dominant view of the community was that SSE in multi-element sensors was analogous to that in single-element sensors, and was mostly or entirely optical in origin [9,10,11,12,13], with some mention of digitization and pixel geometry [9]. Hereafter, SSE specific to multi-element sensors is referred to as multi-element SSE, or MSSE. Note that some older systems did experience SSE-like artifacts of nearby coupling or whole-image offsets dependent on total scene illumination, but these have been effectively eliminated by techniques such as blind subtraction bolometers that have also greatly reduced column noise.

These optical effects could in theory be corrected by measuring the Point Spread Function (PSF) produced by a point source for the specific array and optical configuration. This can be challenging to measure with thermal imaging due to noise unless high object temperatures are used, which may produce a PSF specific to the object temperatures which have a wavelength distribution that differs too much from that of lower temperatures. Budzier and Gerlach examined MSSE in imaging arrays [9] and measured the PSF for higher temperature point sources. They pointed out the artifact was present even in large illuminations and stated “When imaging large objects, such as area blackbodies, not only the edge areas are affected, but also the entire image.” (e.g., see Figure 9 in [9]). To reduce the impact of the observed temperature reduction, they developed a correction based on the Modulation Transfer Function (MTF) derived from this PSF. Schramm et al. [10] developed a convolution kernel based on pyrometer SSSE models, which assumed an optical source when defining kernel weights with an exponential decay by the radius from the center of the kernel. Schramm later compared the convolution and MTF approaches and showed a greater reduction in MSSE seemed possible when using both correction methods albeit noting that the deviation from real data is significant [11], and this same group later improved the MTF method but again noted this method fails to reproduce the magnitude of the artifact and that more work is needed, specifically mentioning scattering as a possible reason for the failure of the MTF method [12]. Both methods can be seen to produce either under-correction in central parts of images and, for the convolution method, over-correction near edges (see Figure 3’s temperature profiles in the upper-right quadrant following convolution correction in [10] for both over- and under-correction in the same line profile and Figure 7 for a fused approach; see also Figures 12 and 13 in [12] for the magnitude of the under-correction and model comparisons). We will show later how a simple line profile of temperatures across the image crossing the center of a source having a simple geometry such as a circular disk can be a powerful tool for characterizing the artifact and visually assessing the accuracy of correction in a manner that translates to arbitrary geometries.

Past methods for characterizing MSSE are limited to empirically searching for the minimum physical size of an object that can be accurately measured [7,8], which is not suitable for obtaining deeper insights on the nature of MSSE, and this focus on “size” of a calibration object may have hindered the characterization of MSSE with other covariates, such as pixel extent. Several recent works have greatly improved the characterization of MSSE [12,14,15,16]. Pušnik and Geršak [14] characterized MSSE using several source geometries (rectangular slits, square arrays) and used larger ROIs and examined the subtended pixel extent. Their use of pixel extent, rather than size or relative size of an object being viewed, is an important enabler for characterizing the artifact. Critically, they reported the pixel extent of the artifact was greater than investigators typically anticipate.

Independently, the author [16] and Mendez et al. [12] used occluding plates, each having a circular cutout of a specified diameter, placed over a calibration source to produce radially symmetric calibration sources of various diameters. This equipment was used to obtain the reduction in measured temperature [16] or radiance [12] measured at the imaged hole’s central pixel from that of the same or similar pixel under maximal exposure to the calibration source (maximal exposure being empirically verified to be larger than the largest size source that results in a reduction in temperature), plotted versus the hole size in inches [16] or percent of maximal object size [12]. Later, Mendez et al. improved [15] upon this by using an adjustable iris to obtain finer-grained measurements.

For an artifact that can be as large as a few degrees, we should expect a much smaller residual from SSSE-inspired corrections. It has therefore become clear there is a sizable artifact and that it may not be entirely due to the same optical effects as SSSE theory would suggest. Regardless of the actual source of the artifact, characterization and better correction are desirable goals and this work is focused on those ends.

For many applications, especially qualitative assessments, MSSE may be ignored as an unimportant detail. However, applications requiring surface temperature accuracy of real-world objects will experience impaired accuracy. Body thermometry with thermal imaging is a good example of an application that is critically dependent on control over MSSE, an issue that has only been raised very recently [14]. To motivate the importance and practical impact of this work, Figure 1 shows the effect of the methods described herein on a thermal imaging system used for body thermometry (image obtained with first system in Table 1 and correction described in Equation (13)). Uncontrolled MSSE can result in under-estimated body temperatures and therefore, it is critical to account for MSSE in body thermometry.

This image was acquired of the author in a room with 22.0 °C ambient air temperature and 47.5% RH (Sensiron SHT41), core (oral) temperature of 36.4 °C measured by direct mode (3 min in sublingual pocket by trained operator) oral thermometry (SureTemp Pro by Welch Allyn, Skaneateles, NY, USA), at a distance of 1.08 m to the subject’s face (measured by time-of-flight sensor, resulting in pixel size on target of 0.94 mm), with a flat background behind the subject and no emissive objects within the room (other than the subject, the camera and a laptop computer). Canthi was identified manually (pixel probes indicate region for surface temperature determination on each side), with estimated core temperature determined using a prior-determined clinical physiologic correction dependent on air temperature, but note that this camera system was not fully qualified for human body thermometry, as this physiologic correction was determined using a different camera system.

A major challenge for validating the accuracy of facial imagery is the lack of ground truth, rendering it challenging to draw conclusions on observations. To help address this limitation, Figure 2 shows the effect of the methods with a 20-spoke Siemens star. Figure 2c shows line profiles of uncorrected and corrected pixels covering the length of one spoke.

In this paper, we will build upon the work of other researchers and use the imaged object extent in terms of pixels illuminated, rather than physical size of the occluding plate’s hole, and the fractional deviation in measured temperature for our characterization and correction methods. Using this characterization, we demonstrate a method to predict the reduction in accuracy for a simple application in terms of pixels on target and the expected spread in temperatures. Finally, we develop an objective correction method that requires only a set of circular-shaped calibration target measurements.

## 2. Materials and Methods

Three thermal imaging systems (Boson 640 and Lepton 2.5 by Flir Systems, Wilsonville, OR, USA and Micro80 by Lynred, Grenoble, France) were used to collect measurements with a single calibration target (4181 Precision IR Calibrator by Fluke Corporation, Everett, WA, USA) with target temperatures denoted by $T_{T}$ with three setpoints of 35 °C, 50 °C, 80 °C; one system was operated with three different lenses that shall hereafter be denoted by the hFOV of each lens (e.g., 23° Micro80 for the system in the third row) (Table 1).

An adjustable iris (2–50 mm Adjustable Iris Aperture by Beufee, Wuhan, China) was attached to a fixture constructed of foam-core posterboard material (1/4” or 6.5 mm thick) to be placed between the imaging system and the calibration target. The optical pathway between the imaging system and calibration target was defined by a supporting platform (same material as the fixture) containing the iris fixture. This platform constrained the angle of incidence to 20 degrees off-axis such that reflections from the iris would originate from a reflection stop frame instead of the IR system’s emissions. This was necessary due to reflections because the iris’s black painted surface was not sufficiently emissive. An emission stop frame was placed in front of the IR system’s optical pathway to cover most of the IR system’s emissions. Each stop frame was constructed of the same posterboard material. This platform further allowed the IR system and its emissive stop to move closer and further from the iris while maintaining the same angle of incidence, to enable tuning of the distance to achieve the best focus prior to beginning the wait for stabilization. The stabilization was assessed by monitoring the sensor’s silicon die temperature as reported by the sensor firmware (focal plane array, or FPA, temperature) and requiring this to change by less than 3 ADC counts per minute. The iris was manually actuated during each acquisition using the ring mechanism. The IR calibration target’s emissions were blocked from the backside of the iris to prevent heating until within a few seconds before each acquisition was initiated. A diagram of the acquisition configuration is shown in Figure 3.

The following checklist was used prior to each acquisition of a set of iris diameters for one setpoint, per camera system:

- Surface temperature around fixture and reflected stop measured within the enclosure with infrared spot-meter;
- Calibration target at setpoint and stable;
- Room temperature stable with no air drifts and ambient temperature and humidity recorded using traceable instruments;
- Focus tuned using an online edge-detection routine and adjustment of distance to target after an initial focus setting;
- Iris heating minimized by blocking radiant heat from calibration target before each acquisition;
- Internal sensor FPA temperature stable for the duration of each acquisition;
- Acquisition time kept short, typically below 20 s, to minimize drifts introduced by exposure to calibration target.

For each setpoint and system, once the configuration was stable, the acquisition was initiated and the iris was stepped between maximum and minimum diameters manually. Acquired images of three different diameter iris settings are shown in Figure 4a–c, with profiles of these three images shown in Figure 4e. The hygrometric conditions should be monitored during the acquisition with traceable instruments. However, due to an oversight, the ambient temperature and humidity for the data acquired in this study were captured with a sensor lacking a traceable calibration chain (SHT41, by Sensiron, Stäfa, Switzerland, ±0.2 °C, ±0.5% RH). The ambient temperature ranged from 22.4 to 24.1 °C and humidity ranged from 24 to 29% RH.

### 2.1. Analysis of Disk Images

Three measurements are required from each disk image: the temperature at the center of the disk $T_{meas}$, the background temperature $T_{BG}$ surrounding the disk, and the size of each disk. Two initial measurements are needed to help localize and refine these measurements: the maximum temperature $T_{maxima}$ and the estimated background temperature $T_{BG-estimate}$. The measurement of $T_{maxima}$ and $T_{BG-estimate}$ are straightforward, while the background is somewhat less straightforward because the region immediately surrounding the disk is also affected by artifact. The background can be measured by first setting a threshold closer to the background, and then growing successive rings outwards from the initial thresholded disk that are progressively less affected and selecting rings far enough from the disk that no further change in temperature is seen, thereby avoiding residual MSSE. The disk size measurements are less straightforward, as the measurement is unavoidably dependent on choice of thresholding used to reduce the impact of partial pixel artifacts, diffraction and MSSE and thereby obtain a best estimate of the true extent of illumination of a disk. These effects can introduce bias in the size measurement especially at smaller disk sizes, but this can be avoided by eliminating the smallest size disks and selecting a threshold closer to the maxima. The tendency to bias due to increasing involvement of partial pixel as size decreases was considered more important to avoid, and therefore, based on earlier measurements of the magnitude of MSSE, a threshold of 90% of the difference should mostly avoid partial-pixel effects while allowing for reduction due to MSSE and diffraction that the size corresponded to illumination extent.

Each disk image was analyzed by first obtaining the maximum temperature as $T_{maxima}$ and an estimate of the background temperature using the measured ambient temperature as $T_{BG-estimate}$. A first mask was obtained by cropping to values above a threshold $T_{threshold1}$ of 10 percent of the difference $T_{maxima}$ and $T_{BG-estimate}$ plus $T_{BG-estimate}$, as shown in Equation (1). Additional morphological operations were used to obtain masks of rings surrounding the disk by dilating repeatedly. A range of ring masks applied to the image having average temperatures within 100 mK of the most-dilated value were selected to avoid any impact from the disk. The temperatures within this set of rings were averaged to obtain $T_{BG}$. A second mask was obtained by cropping to values above a threshold $T_{threshold2}$ of 90 percent of the difference between $T_{maxima}$ and $T_{BG}$ plus $T_{BG}$, as shown in Figure 4e and in Equation (2).

(1) $$T_{threshold1}=0.10\times \left(T_{maxima}-T_{BG-estimate}\right)+T_{BG-estimate},$$

(2) $$T_{threshold2}=0.90\times \left(T_{maxima}-T_{BG-estimate}\right)+T_{BG-estimate},$$

The radius *r* and centroid (x,y) were obtained by fitting an ellipse to the second mask image. Similar radii can be obtained by taking the square root of the number of pixels divided by 2π, which can be useful when the radius is too small to effectively fit with ellipse-fitting; however, in this work, all data with radii less than or equal to 2.75 pixels were not used. The rationale for only using radii greater than 2.75 pixels is the likelihood of diffractive and partial pixel artifact obscuring the results. The value at the center of the disk image was obtained by taking the median of the central pixels as $T_{meas}\left(r\right)$, denoted by $T_{meas}$ in Figure 4f.

For each system and setpoint, the $T_{T}$ used in the analyses was not identical to a blackbody setpoint, because the IR system’s calibrated outputs were insufficiently accurate. Therefore, each $T_{T}$ was determined from the extrapolated maximum output temperature obtained based on the temperatures versus disk size using inverse radius fits empirically selected for linearity with the measured percent deviation defined in Equation (4). The deviation $\Delta (T_{T},r)$ for a disk with radius *r* is defined in Equation (3) as the difference between the setpoint $T_{T}$ and the measurement $T_{meas}$:

(3) $$\Delta \left(T_{T},r\right)=T_{T}-T_{meas},$$

This deviation depends on the target and background temperatures $T_{T}$ and $T_{BG}$, but can be simplified. Earlier work [16] found that the deviation divided by the difference between target and background was invariant to each of these two temperatures. Therefore, we define the percent deviation in Equation (4):

(4) $$Pct\left(r\right)=\frac{T_{T}-T_{meas}}{T_{T}-T_{BG}},$$

$T_{meas}$ for the 35 °C setpoint is shown in Figure 4f above, and the $T_{meas}$ for the 50 °C setpoint is shown below in Figure 5a. The calculated Δ of Equation (3) is shown in Figure 5b, and the calculated *Pct*(*f*) of Equation (4) is shown in Figure 5c.

Earlier work [17] relying on a smaller set of six circular occluding plates identified a 1/r dependence; thus, the Pct(r) was initially investigated assuming it to be linear with 1/r. The higher resolution of the iris method with the Boson data revealed this relationship was not linear. A search procedure over exponents applied to the radius indicated the Boson Pct(r) data was only linear upon application of a power factor to the inverse radius, leading to an alpha parameter and a more general fit:

(5) $$Pct\left(r\right)=\frac{m}{r^{\alpha }},$$

The Pct(r) data were linearly fitted to the inverse radius raised to an empirically determined power factor α for a set of data (or the full set of data for a system) to obtain the slope *m*. This α was obtained by a search procedure to find an α value per data in Equation (5) that produced the best linearity. The best linearity for the Boson system was obtained with α near 0.5, while the best linearity of the other systems was obtained with α near 1.0.

### 2.2. Derivation of Convolution Correction

We now turn to a model of the artifact. A discrete 2-dimensional convolution of weights *w* (of size W×W, e.g., 133 × 133 for 640 × 480 images and 55 × 55 for 80 × 80 and 80 × 60 images, selection of which will be described later) applied to image *I* (w∗I) is hypothesized to produce an additive adjustment to the underlying “truth” image in Equation (6):

(6) $$I_{meas}\left(x,y\right)=I_{truth}\left(x,y\right)+\sum_{i,j}^{W}w_{i,j}\times I_{truth}\left(x_{i-W/2},y_{j-W/2}\right),$$

Note that $I_{meas}$ is the calibrated output image of a system and this additive adjustment would also be present during the calibration process. The purpose of microbolometer calibration is to obtain suitable object temperatures with sufficient accuracy. Microbolometer calibration coefficients and ancillary data, such as the FPA temperature and pixel signal level, are combined to transform a measurement of signal for a given pixel into the estimated scene temperature subtended by that pixel, by removing the influence of non-scene illumination such as that emitted by the optical pathway and the microbolometer itself. A calibration process typically involves the full-field exposure of the system to a calibration target under controlled conditions for several stable system temperatures and several stable calibration target temperatures. An appropriate parameterization of the system is fitted to that data, which can then be used to obtain an output linear with scene temperature on arbitrary scenes. Some calibrations only aim for linearity while others aim for absolute accuracy. Many microbolometers exhibit offset drift that varies across pixels, and an additional correction using an actuated shutter is often used, but this is not expected to alter the hypothesized crosstalk. If this hypothesized crosstalk is present during the calibration process and this process is performed with a full-field illumination, the user of the calibration process may be unaware the illumination for each setpoint incorporates this additional crosstalk, or off-pixel, stray illumination that would then be less present under partial-field illumination. To summarize, MSSE is not seen in full-field uniform illuminations, whereas MSSE artifact in partial-field illumination is a modulation in signal dependent on the difference in partial-field crosstalk versus the full-field crosstalk. Returning to the model, for a full-field illumination where every pixel is exposed to the same object temperature, the convolution condenses to the full-field image multiplied by the constant sum of kernel weights in Equation (7):

(7) $$I_{meas}^{FF}\left(x,y\right)=I_{truth}\left(x,y\right)+I_{truth}\left(x,y\right)\times \sum_{i,j}^{W}w_{i,j}=I_{truth}\left(x,y\right)+I_{truth}\left(x,y\right)\times C,$$

where C is the constant sum of kernel weights. The full-field measured image is however calibrated to produce the expected measurement, so all data obtained from a sensor that is affected by MSSE already incorporates this hidden correction factor, such that $I_{meas}$ is the expected “correct” image for full-field illuminations. Therefore, correction of MSSE must reproduce this $I_{meas}$ by incorporating the $C\times I_{truth}$ factor (or the system must be calibrated differently).

For partial-field illumination, the difference between the image at pixel x, y and full-field illumination produces a subtly different image in Equation (8):

(8) $$I_{meas}^{PF}\left(x,y\right)=I_{truth}\left(x,y\right)+\sum_{i,j}^{W}w_{i,j}\times I_{truth}\left(x_{i-W/2},y_{j-W/2}\right),$$

where the convolution does not trivially condense to a constant due to the presence of different temperatures across the image. The difference between partial-field and full-field illumination produces a deviation image $\Delta \left(x,y\right)=I^{FF}\left(x,y\right)-I^{PF}\left(x,y\right)$, at each pixel (x,y) in Equation (9):

(9) $$\Delta_{truth}\left(x,y\right)=I_{truth}\left(x,y\right)\times C-\sum_{i,j}^{W}w_{i,j}\times I_{truth}\left(x_{i-W/2},y_{j-W/2}\right),$$

(10) $$I_{truth}\left(x,y\right)=I_{meas}+\Delta_{truth},$$

The corrected image can then be obtained by adding Δ to $I_{meas}$ as in Equation (10). Because $I_{truth}$ in $\Delta_{truth}\left(x,y\right)$ is not known, it may be approximated by denoting $I_{meas}$ as $I_{corrected}^{0}$ to produce $I_{corrected}^{1}$ as the first-approximation to the correct image in Equations (11) and (12).

(11) $$I_{corrected}^{1}\left(x,y\right)=I_{meas}+\Delta_{corrected}^{0},$$

This results in an iterative correction, each step *i* using the $I_{corrected}^{i}$ output of the previous in place of the ${\left(i-1\right)}^{th}$$I_{corrected}^{i-1}$ image, as shown in Equation (12) and thereby approach $I_{truth}$.

(12) $$I_{corrected}^{1}\left(x,y\right)=I_{meas}+I_{corrected}^{0}\left(x,y\right)\times C-\sum_{i,j}^{W}w_{i,j}\times I_{corrected}^{0}\left(x_{i-W/2},y_{j-W/2}\right),$$

However, large 2-dimensional convolutions are computationally expensive. Fortunately, it may not be necessary to iterate the convolution portion. Due to the spatial structure of the weights and their slow change over many pixels, the convolution portion may produce a smaller iterative improvement than the non-convolution portion. Therefore, if $W*I_{meas}$ is sufficiently close to $W*I_{truth}$, only a single convolution may be needed, but the $I_{meas}\times C$ component must be iterated to produce a sum of $C^{N}$ terms as in Equation (13). This will be evaluated after obtaining the kernel weights. This sum condenses to the form in Equation (13) where the first $I_{meas}\left(x,y\right)$ is not a convolution but merely the pixel at x, y, and the second $I_{meas}$ is the sole convolution required:

(13) $$I_{corrected}^{N}\left(x,y\right)=I_{meas}\left(x,y\right)\times \sum_{k=0}^{N}C^{k}-\sum_{k=0}^{N-1}C^{k}\times \sum_{i,j}^{W}w_{i,j}\times I_{meas}\left(x_{i-W/2},y_{j-W/2}\right),$$

Next, this treatment is applied to the class of images of circular disks having a uniform target temperature $T_{T}$ surrounded by a uniform background temperature $T_{BG}$ and the deviation is only considered at the pixel located in the center of the disk. These simplifications enable an analytical solution to obtain the kernel weights. Critically, we can obtain the $T_{truth}$ from the full-field or largest disk image or the fit of $\Delta (T_{T},r)$ extrapolated to the maximum radius.

Briefly returning to 2-dimensional convolution, the kernel is applied in a sliding-window fashion to every pixel in the image, as shown in Figure 6a. Our hypothesis, based on the apparent radial symmetry of the artifact and its dependence only on differences in temperatures to that pixel, is that the artifact may be approximated by applying the convolution weights to the pixels surrounding that one pixel. Therefore, we can restrict our view to only weighted sum centered on the pixel in the center of the disk, rather than a full-image convolution, which simplifies the scenario. Under this simplification, the resulting artifact would be the sum of only those weighted pixels, as shown in Figure 6b.

Under these simplifications, the deviation at the center of the disk at coordinates $c_{x},c_{y}$ having temperature $T_{T}$ surrounded by a background temperature $T_{BG}$ is modeled by a convolution applied to the difference in Equation (14):

(14) $$\Delta \left(c_{x},c_{y}\right)=\sum_{i,j}^{W}w_{i,j}\times \left[T_{T}-T_{BG}\left(x_{i-W/2},y_{j-W/2}\right)\right],$$

where for this single pixel, there are no contributions from the constant values in the disk itself, but only the constant valued background pixels where $x_{i},y_{i}$ are outside the disk, up to the image dimension, here taken as *X*. Assuming radial symmetry of the kernel weights, the convolution may be taken in 1-dimensional polar coordinates over the pixel distance from the center of a disk having a radius R as Equation (15).

(15) $$\Delta \left(R\right)=\sum_{r\geq R}^{X}2\pi rw_{r}\times \left[T_{T}-T\left(r\right)\right],$$

where

$$T\left(r\right)=\left\{\begin{array}{cc} T_{T}, & \mathrm{if}\;r<R\\ T_{BG}, & \mathrm{if}\;r\geq R \end{array}\right.$$

Taking the derivative of the deviation as a function of radius produces Equation (16):

(16) $$\frac{d\Delta (R)}{dr}=\frac{d(rw_{r})}{dr}\times 2\pi \left(T_{T}-T_{BG}\right),$$

which can be compared to the derivative of the empirically fitted Pct(r) of Equation (5) to produce Equation (17):

(17) $$\frac{dPct(r)}{dr}=-\frac{m\alpha }{r^{\alpha +1}},$$

which can be converted to the temperature deviation Δ(R) by multiplying by the difference in temperatures $\left(T_{T}-T_{BG}\right)$; the product can be equated with the derivative in Equation (16) to give the solution for the kernel weight at a radius R from the center of the convolution kernel in Equation (18):

(18) $$w_{r}=-\frac{m\alpha }{2\pi r^{\alpha +2}}.$$

This approximation is used to obtain the kernel weights for the correction procedure (note that this retains the term 2π). Specifically, the radius is replaced with the indices of the regular array i,j in Equation (19):

(19) $$w_{i,j}=-\frac{m\alpha }{2\pi r_{i,j}^{\alpha +2}},$$

where $r=\sqrt{{\left(\left(W-1\right)/2-i\right)}^{2}+{\left(\left(W-1\right)/2-j\right)}^{2})}$ for r>0 for a kernel of size W×W. Finally, the correction is obtained by applying this kernel to thermal images as in Equation (13).

## 3. Results

### 3.1. Iris Data

#### 3.1.1. Percent Deviation Versus Radii

For each system in Table 1, *Pct*(*r*) was found to be independent of each of $T_{T}$, as shown in Figure 7. See Appendix A for *Pct*(*r*) plots for each system, at each setpoint $T_{T}$.

The initial results motivating this work led to the hypothesis that MSSE could be corrected with a single alpha and slope per optical configuration (regardless of target and background, for a reasonable range of both). However, these estimated parameters are impacted by the presence of noise and signal drift. Different alpha and slope parameters can lead to much improved residuals for a specific scenario. Improved measurement techniques may produce more consistent results in future. It is possible the best results may indeed be reached only with parameters specific to a particular set of target and ambient temperatures.

#### 3.1.2. Percent Deviation Versus Inverse Radii and Fit

A linear fit of the data for each system with Equation (5) revealed good linearity and produced similar slope factors for each setpoint for a system, as shown in Figure 8a–c and Figure 9a–c. Applying this fit to the inverse radii data produced strong linear correlation to the *Pct*(*r*) with slope and r-value indicated in the legend for each plot. The fitted slope factors were comparable across the three $T_{T}$ datasets within each system.

### 3.2. Characterization of MSSE

*Pct*(*r*) in Equation (5) as a function of m and α can be used to predict practical impact for an application. However, the *Pct*(*r*) fits can only be used for a worst-case, binary scenario of a warmer region of interest surrounded by a cooler background (or vice versa). This requires estimates for $T_{BG}$ and $T_{T}$ and the pixel extent of the target’s illumination on the FPA to estimate the deviation $\Delta_{MSSE}$. Note that pixel extent is equivalent to the diameter, and therefore, radius *r* is half the pixel extent.

#### 3.2.1. Experimental Impact for Simple Scenes

In most applications, the pixel size is only indirectly obtainable through the directly known sensor’s iFOV, distance to target, and size of physical feature; therefore, we examine the impact by translating *Pct*(*r*) to Pct(distance) for two of the systems in Table 1 for a specific application, body thermometry, in a specific worst-case scenario.

Body thermometry with thermal imaging involves the acquisition of surface temperatures of the inner canthi and applying a clinical correction incorporating air temperature to obtain a body temperature estimate [18]. In this example, we only consider the accuracy of the surface temperature measurement of the canthi, and consider a worst-case scenario where the human subject is wearing a near-total face covering at ambient temperature that exposes only the eyes and nasion. In this scenario, the exposed region of skin is close to the canthi temperature of 35 °C and is approximately 40 mm across and the background clothing around the exposed skin is 20C. While typical face coverings are likely to be warmer than ambient, for the sake of this example, we proceed as if the remainder of the illumination is a uniform 20C and the face region is a uniform 35 °C. Finally, the required spot size on target is 5 mm. The tangent of the pixel iFOV for a system times distance *D* produces the pixel size as a function of distance, and the number of pixels covering a spot size having one dimension L is then L/pixel size, with radius being half of this, allowing the insertion of these values in place of radius. From these inputs and the fitted *Pct*(*r*) equations for two selected systems, we can convert *Pct*(*r*) to the deviation Δ expected for the target and background temperatures ($T_{T}$ and $T_{BG}$), distance *D* to target, and iFOV as shown in Equation (20) to obtain Figure 10.

(20) $$\Delta \left(T_{T},T_{BG},D,iFOV\right)=\left(T_{T}-T_{BG}\right)\times m\times {\left(\frac{2\times D\times \tan(iFOV)}{L}\right)}^{\alpha },$$

The lines in blue refer to the first system in Table 1, and the lines in red refer to the last system in that table, costing an order of magnitude less than the first system. The lower-cost system does have a shorter required range to achieve the required spot size (just under 1 m), but is able to achieve nearly half the deviation without any correction applied. We point out the shape is related to the fitted alpha, and engineers unaware of the MSSE artifact may attempt to measure and fit a distance-dependent correction to attempt to ameliorate the impact, but would observe highly variable efficacy across subjects and especially as the environmental temperature changes. If the person’s facial structure is typically flushed, the reduction will vanish and the correction may introduce false positive abnormally high predicted body temperatures. Other applications, such as monitoring machinery for changes in temperature, will suffer similar challenging deviations arising from cooler or cooler-appearing reflective surfaces surrounding a warm bearing or electrical connection.

It should be pointed out that a maximum of 1 mm pixel size-on-target is recommended by the international consensus standards covering thermographs for human febrile temperature screening; however, we point out this recommendation was made without considering the recently reported MSSE effects. It is possible that a larger pixel size is acceptable, and our past work has indicated the upper limit of 5 mm [19] if MSSE is well-controlled [16]. MSSE affects facial imaging due to physiology and ambient conditions. In many indoor scenarios, the ambient temperature is physiologically neutral or cool, which results in varying facial thermal structure from person to person, with many individuals developing colder nose and cheek regions, which therefore produce reductions (note the artifact can symmetrically produce increases, if a hotter source is nearby the pixels of interest) in measured surface temperatures and abnormally low predicted body temperatures. Given the fact that past recommendations were made without a correction or characterization of MSSE, prior assumptions such as required pixel size should be re-examined. Further discussion of required spot size is beyond the scope of this work.

#### 3.2.2. Experimental Impact for Complex Scenes

If either the target area or background areas are non-uniform in temperature, the impact can only be obtained by application of the correction kernel to data, which may not translate easily across sessions of a given scene, because the detailed structure of the scene is often session-specific. For example, the same human face often shows large changes in temperature in sessions acquired only an hour apart, and different human faces can be expected to present even greater diversity than this. Therefore, while the impact of MSSE in scenarios with relatively constrained diversity in surface temperature structure, such as energized equipment or rotating equipment, may be reasonably well estimated by assessing field data in one or only a small subset of conditions, scenes of more diverse surface structures, such as human faces, may require multiple samples of a human population and at multiple sessions of each face before a reliable range of estimated impacts may be made. The estimation of impact in complex scenes is beyond the scope of this work.

### 3.3. Correction Kernels

The kernel was generated by applying the α and the fitted slope in Equation (19). Note the center of the kernel is zero because the weights are only relevant for surrounding pixels. The kernel weights fall off rapidly as distance from the center increases, as shown in Figure 11.

Due to the smoothness of the kernel and its decay being faster than the harmonic series, the coefficients summed at a radius are guaranteed to converge but it is important to assess whether the convergence is rapid enough for a practical cut-off in kernel size. We must point out the kernel weight values do not depend on the size of kernel; rather, the choice of size results in truncating from the largest possible kernel, which in turn is dependent on the image size. Therefore, size of kernel used is a tradeoff between accuracy and efficiency. The size of kernel required for an application can be obtained with similar information as used to obtain the distance dependence in Figure 10. If one has the worst-case difference in temperatures and can assume the size of object in pixel extent will be smaller than the smallest kernel selected, the impact can be determined by comparing convolution of the largest empirically derived kernel and centered truncations of this same kernel having a range of smaller sizes, applied in each case to a synthetic circular disk of the expected size. The value in the center of the disk for each size kernel can be compared with the largest kernel, and the smallest size where the residual is not greater than the desired accuracy gives the smallest acceptable kernel size. Unfortunately, this is dependent on the details of the scenario so it can only provide the worst-case required minimum kernel size.

The smaller α required for the Boson produces a slower convergence to zero, and therefore, achieving a given accuracy would require a larger kernel than a system having a larger α. The slope of the fit affects the global scale (applies to all kernel parameters equally), but the rate of convergence can be more important. For the results in this paper, a kernel size of 55 × 55 was used for the Micro80 systems and a kernel size of 133 × 133 was used for the Boson. The kernels used in this study are shown in profile to illustrate the relative weights near the central pixel.

### 3.4. Correction Results

Applying the convolution model in Equation (13) to the data produces a spatial pattern matching the deviation observed versus a full-field illumination. The difference between the corrected image and the uncorrected image is shown below as an image and profiles across the disk region in Figure 12 for the same disk regions shown in Figure 4.

This was applied to each image to produce the $T_{corrected}$ image, which was then analyzed to obtain $Pct_{corrected}$(r). This was compared with uncorrected $T_{meas}$ and *Pct*(*r*). The correction is assessed over the range of radii down to 2.75 pixels and as high as the data was collected. Note that we did not specify an upper stopping radius, because $T_{meas}$ (or $T_{uncorrected}$) approaches $T_{corrected}$ at higher radii. Furthermore, the assessment across radii does not directly translate to impact on an application, rather this should be viewed as preliminary work on a potential approach to a new characterization of the correction. Properly specifying a characterization for complex scenes will require replication and study by other experts in the field and is beyond the scope of this work. The impact here is assessed in $T_{meas}$ and $T_{corrected}$ for: (1) consistency across radii using the standard deviation (denoted as σ) and (2) for maximum deviation from an ideal measurement using the difference between the upper and lower 5 percent quantiles (denoted as R for range). Quantiles were chosen to limit the impact of outliers, which were present in the lower-resolution data.

The $T_{meas}$ and $T_{corrected}$ are shown in Figure 13 and Figure 14 below, with *Pct*(*r*) for the same and for all three $T_{T}$ setpoints. The correction produces measurements that do not vary as a function of illuminated pixel size, although for the smaller illumination sizes, the corrected data shows increasing deviation.

The standard deviation (σ) and range between $T_{meas}$ and $T_{corrected}$ at the 95th percent and 5th percentile (range, or *R*) are shown along with the slope used for each system and $T_{T}$ setpoint in Table 2 below, showing the correction reduced peak-to-peak variability by between a factor of 2 for the worst-performing system (Lepton) and a factor of 8 for the best-corrected system (Boson).

It is important to note the $T_{corrected}$ results apply to the center of the illuminated disk, which does not account for possible deviations in other parts of the disk. This was assessed by examining the uniformity of uncorrected and corrected profiles across disks of a range of sizes, demonstrating that the correction produces more consistent results across different size disks and along each profile as shown in Figure 15 to be compared with profiles in [10,12].

### 3.5. Exponent Search Process

The α and slope *m* that best accounts for the iris data may be obtained by a simple grid search with Equation (5). Specifically, *Pct*(*r*) data is fitted with linear least squares to the inverse radius raised to each of a grid of α values ranging from 0.35 to 1.5 (1000 points) and the residual sum for each value is collected. This produces a smooth curve for each dataset, each having a minimum which obtains the best α and corresponding slope *m* to be used for that system. The parameterization of *Pct*(*r*) is not overly sensitive to the α, as small perturbations of it can produce empirical fits having similar-appearing linearity. Figure 16a shows both 35 °C and 50 °C data have minimums at 0.5 and 0.48, respectively, while 80 °C data obtained a lower minimum of 0.42. Figure 16b shows 80 °C data plotted versus radii raised to the selected 0.5, while Figure 16c shows the same with alpha of 0.42 (the value obtained by the search for the 80 °C data).

Due to the smooth fall-off in artifact as distance from the pixel of interest increases, the correction is fairly robust to small or even moderate changes to α (note, however, that slope must be obtained for each α). Figure 16d shows the impact of an intentionally incorrect α with Equation (13), which can still provide good correction down to pixel radii of 20, before exhibiting over-correction. This may be suitable for some applications, but reliance on a suboptimal correction introduces risks in real-world applications that rely strongly on accuracy, such as body thermometry.

### 3.6. Examination of Iterative Correction

One consequence of the correction equation is the need for iteration, raising concerns about convergence of this iteration. Figure 17 shows convergence of the simplified Equation (13) at N = 4, with minimal change beyond 4. Note that the i = 5 correction of Figure 17a varies less than 150 mK but does exhibit some structure, which may be due to the subset of data selected. The data used were isolated images so the results would clearly indicate only the impact of iteration, specifically, the same images as used to obtain a range of profile data, rather than the full set with far more datapoints.

### 3.7. Focus Effects

The MSSE phenomenon may arise from a number of causes, including optical effects that are known to result in spreading signal over adjacent pixels. Fraunhofer diffraction in traditional optical focusing leads implies a limit for the smallest pixel pitch for a given wavelength of light and aperture size N (f-number in Table 1) at p=1.22λ×N. By Wien’s displacement, the peak wavelength emitted from a 308 K target is 9.4 μm, resulting in an Airy disk radius of 11.4 μm. This is too small by at least an order of magnitude to account for the MSSE effects in this work.

An often larger source of signal blur is optical defocus, or a focus setting that does not produce the smallest circle of confusion under the optical system’s geometrical constraints and distance to target. This blurring across adjacent pixels might naively be expected to look much like the MSSE effects documented here and elsewhere. Many users of the technology may erroneously consider MSSE to be simply a matter of operator error in setting focus and therefore disregard the issue of MSSE altogether. To help disambiguate defocus from MSSE, we examined how blur caused by defocus related to the MSSE effects of an optimally focused system by intentionally defocusing the Boson and acquiring $T_{meas}$ data with the iris system. The extent of defocus was assessed by measuring the width of the transition between a heated target and the background as compared to the step function that describes the ideal case. Data was acquired at focus settings producing widths of 1 (ideal focus), 3, 6, and 5 (width of 5 was acquired with defocus in the negative plane versus the data having width of 3 and 6). Figure 18 shows that with this range of defocus, the *Pct*(*r*) data is rougly comparable.

### 3.8. Notes and Limitations

#### 3.8.1. Computational Complexity

The application of two-dimensional convolution to an image can be computationally prohibitive for naive convolution, where naive means the implementation proceeds exactly as defined by sum over weights, moving across the image. For example, a 133 × 133 convolution applied to the entire 640 × 512 image of the Boson would require $133^{2}\times 640\times 512$, or 5.8 B operations, which is prohibitive for most applications. Several alternative approximations to the convolution exist, such as atrous, downsampled, separable or Fourier transform convolution. Due to the smoothness of the kernel, good results may be obtained by applying an atrous convolution that skips every 2–4 pixels, or alternatively, the image may be downsampled by 2–4× and an atrous skipped (and therefore smaller by the same factor) kernel applied before upsampling the result, reducing computational burden by 16–256 fold (many compute systems possess image scaler peripherals that can apply up or downsampling at lower cost and without involving the main processor). Separable convolution requires the kernel to be decomposed, and unfortunately, these kernels’ weights do not separate sufficiently well, otherwise this would be an attractive option. Finally, Fourier transform convolution reduces the cost to a small multiple of that of the transform itself (twice on image dimensions and precomputed one time on the kernel) and $133^{2}$ multiplications. Furthermore, the Fast Fourier Transform (FFT) has a computational complexity that scales as the logarithm of the number of pixels. The process is straightforward, first applying a forward two-dimenstional FFT, then multiplying the central grid of the result by a transform of the original kernel and then applying the inverse-FFT. The most attractive, due to both speed and accuracy is the FFT-based convolution, which has minimal deviation from the naive convolution.

#### 3.8.2. Threshold Dependence

The disk size measurement is affected by choice of threshold, due to the edge effects present from a combination of partial pixel averaging, diffraction and MSSE. This may introduce systematic biases in the empirically fitted *Pct*(*r*)’s slope and α, which could introduce or obscure curvature as a function of radius raised to the power α. It may be possible to obtain size measurements after applying the MSSE correction, but this introduces additional questions as to whether the obtained fits were then biased towards obtaining the conclusions relied upon, and therefore, this was not examined further.

#### 3.8.3. Potential Interference of Spatial Correction with MSSE

For best interpretability, any uncontrolled corrections applied to the data should be eliminated or minimized. The Boson system used in this study normally operates with a temporal filter and a scene-based Non-Uniformity Correction as part of the built-in correction pipeline, and both of these were disabled for all acquisitions because these corrections will affect the results, especially the scene-based Non-Uniformity Correction. The author cannot confirm whether the Lepton performs any temporal or spatial filtering but there are entries for these filters in the documentation for the sensor; however, those entries insufficiently describe the corrections or whether they are used, and more importantly, it does document that those processing functions cannot be configured by the user. If the Lepton is performing two-dimensional convolutions to the image data, it would alter the appearance of MSSE in unexpected ways. Frame differencing implies some temporal filtering is implemented, but this is not conclusive. It is even less straightforward to detect spatial filtering. If spatial filtering is applied, it is likely these results would be modulated.

The Micro80 used in this study was under the full control of the author’s electronic readout circuitry design, firmware and calibration chain and did not have any on-board filtering, although the calibration was optimized for the 23° hFOV lens, which affects the scale of the obtained $T_{meas}$ for the 25° and 39° hFOV lenses.

#### 3.8.4. Stability of Sensor

The thermal stability of the Silicon die (FPA temperature) and optical pathway during acquisitions is critical for stability of the gain and offset, but the Lepton and Micro80 systems have an additional source of system drift due to the use of a plastic housing between the FPA and the lens assembly. Figure 19 shows the optical pathway of the Micro80. We refer to optical pathway to include all surfaces that contribute non-scene signal to the microbolometer array. The three views of the Micro80 show that while the lens assembly is thermally conductive, being composed of Aluminum, it is mated to a plastic housing that is a poor thermal conductor. This requires more time before a given level of stability and an unknown level of deviations as the optical assembly equilibrates incompletely to the FPA temperature, primarily through the air pathway and the Silicon window shown in the bottom right. This plastic housing greatly impacts stability, regardless of the stability of the FPA temperature, because of its low thermal conductivity. The Lepton incorporates gold-plating on the inside of the plastic housing, but in practice, this does not much improve stability due to the thinness of the plating and the consequence of multiple internal reflections and corner cavities. The Lepton was observed to have higher temporal noise and drift than the other systems. The Micro80’s readout circuit was designed so the microcontroller was located a significant distance from the FPA, specifically to reduce heating impact from the readout microcontroller, whereas the Lepton incorporates a microcontroller directly integrated under the FPA, as shown on the backside of the Lepton in Figure 19, resulting in a large heat flow directly into the FPA. The Boson experiences a very long tailed slow increase in FPA temperature that never stabilized to the desired extent due to the large heat produced by the Myriad 2 processor, which consumes (and must dissipate) several hundred milliwatts, incorporated in the same housing as the sensor and optical assembly.

For best results, avoid systems that rely on plastic housing in the optical cavity and which integrate significant processing capability in proximity to the FPA. However, it is likely that manufacturers will continue to use such housings at the low and mid-resolution ranges due to manufacturability and cost considerations. Furthermore, they are likely to continue to incorporate the readout electronics near the FPA to improve size and modularity constraints, as well as to include increasingly complex postprocessing functions for market differentiation.

Finally, focus effects are known to produce signal deviations through blurring and some may erroneously consider MSSE to be primarily a consequence of poor control over focus. Focus effects were examined and found to generate the same *Pct*(*r*) behavior at radii greater than the detectable blur in pixel size. Figure 18 shows that the *Pct*(*r*) is at least mostly independent of defocus. Nevertheless, acquisitions should attempt to optimize focus in order to obtain reliable data at the smallest radii.

## 4. Discussion

This paper has characterized SSE in multi-element sensors (MSSE) in a manner that implies a distinctly different phenomenon than the Fraunhofer diffraction model. Furthermore, we have confirmed other recent findings [12,14], showing that MSSE hinders accuracy to a much greater extent than is appreciated by most researchers, manufacturers, and users. In fact, the MSSE effect can be an order of magnitude greater than the required accuracy for body thermometry, which implies MSSE could explain the wide variation in accuracies reported with facial thermal imaging [20]. This issue is important because MSSE is presently missing from various standards that require high accuracy from thermal imaging, in particular infrared body thermometry and thermographic febrile screening.

We found that the MSSE effect can be analyzed in terms of illumination extent and as proportional to the difference in temperatures present in Equation (5). The *Pct*(*r*) Equation, for each particular system we examined, produces results that are independent of each of the disk and disk-adjacent temperatures but rather depends on the difference between these. This is a novel way of analyzing the artifact, although there may well be second-order effects and alternative Equations that produce better results using radiance, especially as the wavelength intensity distribution shifts at higher and higher temperatures.

We further linked the size of a circular disk of illumination to pixel distance from the central pixel, and found the effect at the center of a disk can be fitted as a linear relationship to the inverse radius raised to a low power, which again is independent of the target and background temperatures. This fit equation enables useful and accurate predictions of impact of the MSSE on measurements for various simple imaging scenarios such as binary scenes that have temperatures that fall predominantly in two bins.

Based on the radial symmetry suggested by the disk measurements, we developed a convolution model of the artifact in pixel coordinates surrounding the center of the kernel and mathematically linked this to the measured dependence on pixel illumination size, and thereby developed an objective method of obtaining kernel parameters that mirror the artifact. This is the most important contribution of this work. Until now, various models based on Fraunhofer diffraction, MTF measurements, and empirical fitting have been proposed but none have had the correction power of this approach and none have yet enabled an objective determination of the correction based solely on a set of MSSE measurements.

The radial symmetry of circular apertures simplified the scenario sufficient to enable a mathematical treatment, and an adjustable iris was essential for collecting fine-grained data with less well-behaved sensors (e.g., quickly enough to ignore drift, apparatus heating, etc.). However, drawbacks included the low emissivity of the iris, heating of the iris once exposed to a target, and variation in temperature between the iris and the surrounding enclosure. Another shortcoming for all but one of the systems studied was that the drift evident in the lower-resolution sensors led to noisier data. Additionally, the chamber was not enclosed on top, and room temperature control was only manually monitored and room air conditioning was manually disabled for each acquisition, affecting the reflected temperature off the iris. Most importantly, more sensors in more varied conditions should be examined. Past work indicated no impact from FPA temperature, but past work was limited by the use of a small set of occluding plates. A new environmental enclosure is being constructed to re-assess this potential covariate.

Microbolometer calibration can be performed in a variety of ways but the general objective is to obtain an offset and gain for a given FPA temperature such that the signal level can be transformed to a value linear with the scene temperature. Some sensors can be calibrated directly between scene temperature and signal level, if the required range is narrow, such as with body thermometry, but for wider ranges, it is generally preferable to perform calibration using scene temperatures converted to radiance. Pixel crosstalk causing MSSE was considered by the author to not interact in a complicated manner with calibration but this may not be the case. Some adaptive calibrations, such as scene-based Non-Uniformity Correction (NUC), likely would interact less predictably in the presence of MSSE and when attempting to characterize MSSE; however, this can be ameliorated by simply applying the MSSE correction prior to scene-based NUC. Simple offsetting methods, such as the use of an ETRS as used in thermographic febrile screening, will be critically sensitive to the size of the ETRS used as well as the spatial distribution of temperatures on a subject’s face, but again, correction for MSSE prior to offsetting would reduce these errors. Given the effect sizes seen in commercial systems, an evaluation of MSSE should be incorporated into the international consensus standards for febrile thermographic screening and body thermometry (if performed with thermal imaging), as we have previously pointed out [19]. Future work should examine whether and how this crosstalk varies with scene temperature and how this could be inserted earlier into the calibration chain to assess for effects on gain, which may in turn alter the MSSE effects reported in this work.

Previously studied mechanisms for MSSE include electronic crosstalk, stray light from internal reflections and optical physics including diffraction, aberration and scattering, whereas this correction is entirely empirical in nature. It presumes there is some coupling between pixels that is longer-range than diffraction or optical modulation could account for, but this is unsatisfactory. Our previous work on the artifact [16] examined two amorphous Silicon detectors from the same manufacturer having 17 micron and 34 micron pixel sizes, and noted the artifact had the same spatial size in pixels in both sensors (0.58 mm in spatial extent in pixels). This, combined with the minimal dependence of optics with the 17 micron system [16], led us to propose the artifact may arise from within the microbolometer itself, speculating that the semi-transparent Silicon base could allow thermal light to couple via the membrane supports into the die, scatter or reflect off the backside of the die and producing a penumbra of light that then couples into adjacent pixel support structures. This could be tested by intentionally blinding isolated pixels without affecting their sensitivity to induced temperature; however, it would be challenging to obtain an appropriate gain for blind pixels and furthermore requires the support of industry. Nevertheless, it would be useful to measure the presence or absence of signal changes in the blinded pixels as nearby illumination was altered.

Predictive maintenance with LWIR in some cases involves tracking object temperatures over time. For example, rotating machinery have a variety of distinct failure modes, which can be identified some time before actual failure using temperature trends at specific locations. A common accuracy specified for industrial thermographic cameras is the larger of ±2 °C or ±2% of the object temperature. However, MSSE can produce deviations comparable to or even greater than this, depending on the hotspot size and the temperature of the adjacent objects, which in colder conditions can produce a much larger spread in temperatures than those used in this work. Therefore, the presence of MSSE may be impairing the diagnostic power of predictive maintenance with LWIR. Research in this area should examine the impact of MSSE.

Body thermometry with LWIR has a highly divergent literature, with some researchers reporting poor reproducibility with the same devices that others claim can perform as well as direct thermometry. We have shown how the MSSE artifact will produce a variable artifact that has until recently defied predictive attempts—the artifact will depend on how physiologically frigid subjects are feeling and how recently they have experienced cooler air temperatures. Thermal images of a subject may show a cold nose and cheeks at one timepoint and a fully warm face at another timepoint, which can result in a large difference in measured surface temperature due to this MSSE effect. That means one study may well obtain good results and the next could observe poor results, dependent entirely on covariates most researchers are not aware of and therefore could not have considered. Ultimately, LWIR imaging for body thermometry is unreliable unless the MSSE artifact is controlled such that it is not the dominant contributor to inaccuracy. Furthermore, it is essential that the residual impact of MSSE is assessed in a manner that is meaningful to the application. More work is urgently needed to further develop and incorporate the characterization of residual MSSE into the various medical standards.

## 5. Conclusions

The findings of this work are as follows:

1. MSSE is a microbolometer array artifact that can cause long-range pixel effects;
2. MSSE can be characterized by its dependence on the difference between that pixel’s value and an inverse power-of-distance weighted sum of those seen at neighboring pixels;
3. MSSE measured with circular illumination patterns can be linked to a convolution model;
4. MSSE can be corrected to acceptable limits with this convolution-based approach;
5. Consensus standards covering applications affected by MSSE should be re-evaluated.

Most importantly, MSSE can impair accuracy by a far greater degree than is commonly recognized. Expert committees responsible for international consensus standards for medical devices urgently need the community’s help in confirming, refining, or refuting this work, and in developing a means of assessing whether a given system has controlled MSSE sufficiently to meet the required accuracy for real-world thermographic body thermometry.

## Figures and Tables

**Figure 1:**
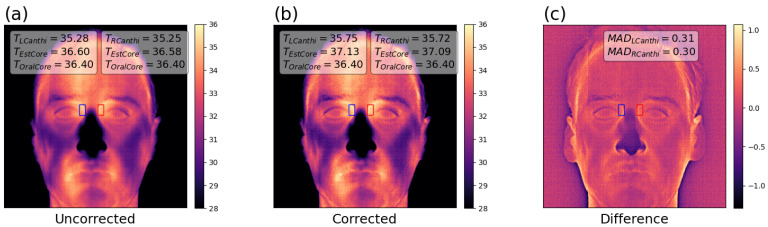
Motivation: demonstration of impact on body thermometry. (**a**) Face image calibrated and offset with in-plane External Temperature Reference Source (ETRS) but otherwise uncorrected, with surface temperature measured at canthi and core temperature estimate by correction with air temperature. (**b**) Same image after MSSE correction with methods described in this paper, with surface temperature measured after MSSE correction and core estimate revealing elevated body temperature. (**c**) Difference between corrected and uncorrected image assessed as Mean Absolute Difference (MAD).

**Figure 2:**
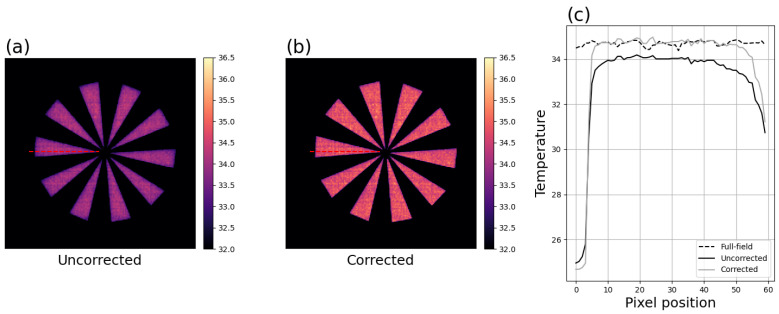
Motivation: demonstration of impact on 20-spoke Siemens star image over 35 °C blackbody. (**a**) Uncorrected image with dotted line indicating pixels of interest. (**b**) Same image after MSSE correction with methods described in this paper, same display scaling, with dotted line indicating same region. (**c**) Pixel profile of indicated pixels and the same pixels from a full-field image acquired within 5 s after the star image.

**Figure 3:**
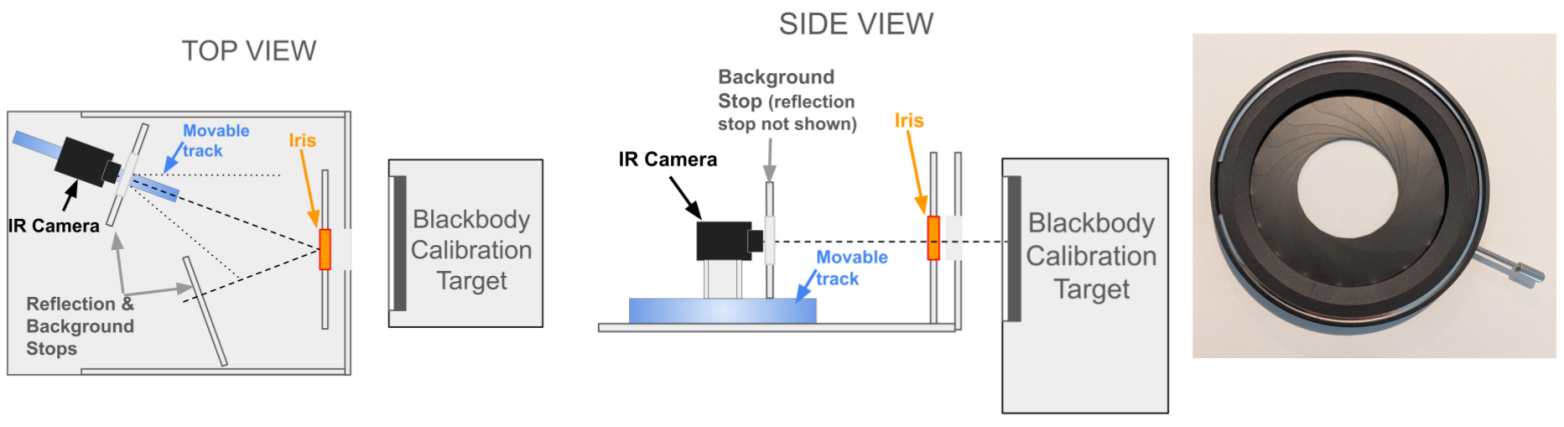
Experimental setup with top and side views of configuration of calibration target, camera, iris and supporting platform; inset to right shows close-up of iris.

**Figure 4:**
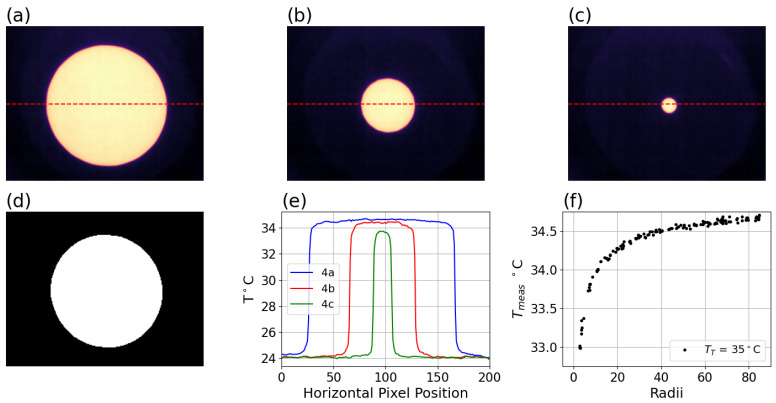
Schema of iris data and disk image analysis methods. (**a**) Large iris radius image crop for 35 °C calibration target setpoint, profile described by dotted line through center of each, profile data is shown in blue in (**e**). (**b**) Medium iris crop, profile through center shown in (**e**). (**c**) Small iris crop, profile through center shown in green in (**e**). (**d**) Iris crop from (**a**) masked for the purpose of fitting radius and centroid. (**e**) Profiles across center of illuminated regions of (**a**–**c**), showing deviation at edges and center, despite numerous pixels on target. (**f**) Measured center temperature $T_{meas}$ versus radius for a full set of iris data at $T_{T}$ = 35 °C. All data is for Boson system in Table 1.

**Figure 5:**
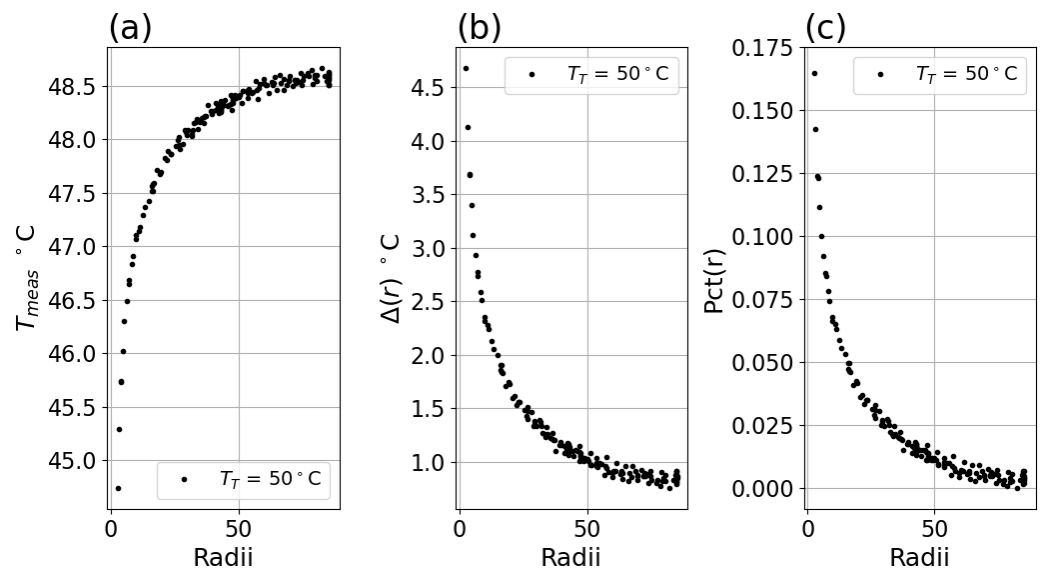
(**a**) Measured center temperature $T_{meas}$ versus radius for $T_{T}$ = 50 °C. (**b**) Δ(r) for $T_{T}$ = 50 °C. (**c**) *Pct*(*r*) for $T_{T}$ = 50 °C. Data in this figure was for Boson system in Table 1.

**Figure 6:**
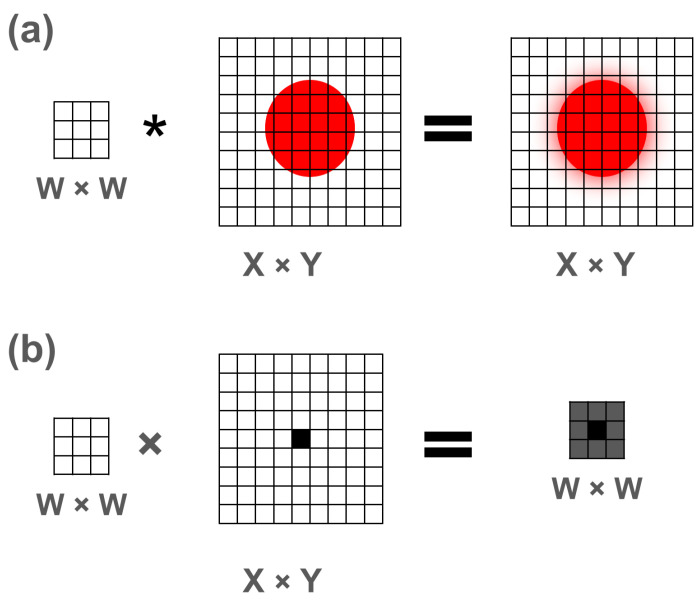
(**a**) Schema of 2-dimensional convolution W∗I, in this case a 3 × 3 kernel applied to every pixel of a 9 × 9 image, producing, in this case, a blurred output 9 × 9 image, with red representing a higher intensity disk. (**b**) Selective application, or W×I(x,y) (note this second form uses × instead of ∗ to indicate our intent of only considering the center of the convolution), of 3 × 3 set of kernel weights to the pixels surrounding a single chosen pixel, denoted here as black, intended to represent the pre-summation terms of the convolution at a single pixel at the center of an illuminated disk.

**Figure 7:**
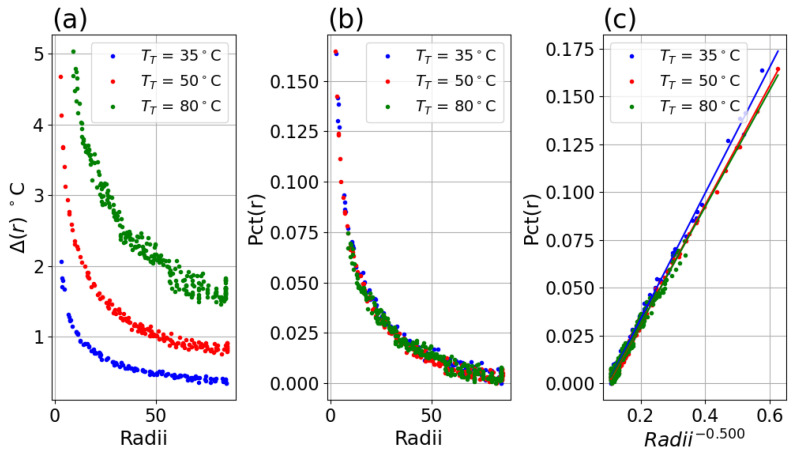
(**a**) $T_{meas}$ versus radii for $T_{T}$ = 35 (blue), 50 (red) and 80 (green) °C setpoints. (**b**) *Pct*(*r*) versus radii. (**c**) *Pct*(*r*) versus radii.

**Figure 8:**
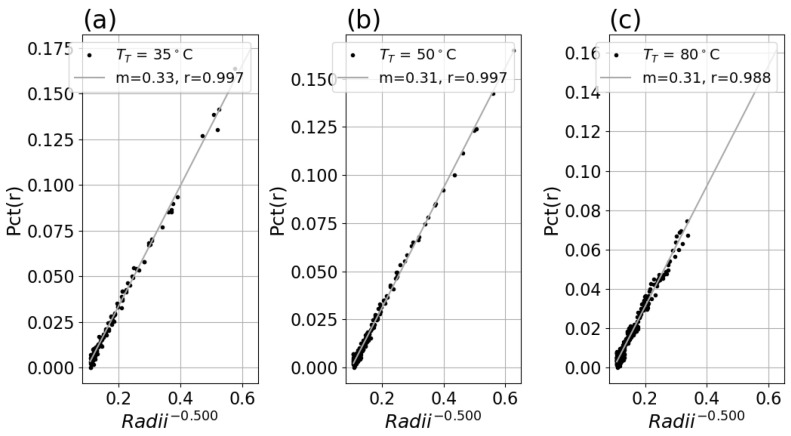
(**a**) *Pct*(*r*) versus $r^{-0.5}$ for Boson system with $T_{T}$ = 35 °C setpoint, with fitted slope and linear correlation in the legend. (**b**) *Pct*(*r*) versus $r^{-0.5}$ for $T_{T}$ = 50 °C data. (**c**) *Pct*(*r*) versus $r^{-0.5}$ for $T_{T}$ = 80 °C data.

**Figure 9:**
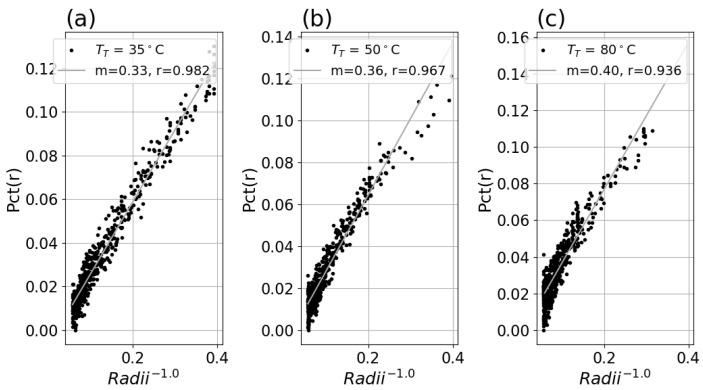
(**a**) *Pct*(*r*) versus $r^{-0.5}$ for Micro80 system and 23° lens with $T_{T}$ = 35 °C setpoint, with fitted slope and linear correlation in the legend. (**b**) *Pct*(*r*) versus $r^{-0.5}$ for $T_{T}$ = 50 °C data. (**c**) *Pct*(*r*) versus $r^{-0.5}$ for $T_{T}$ = 80 °C data.

**Figure 10:**
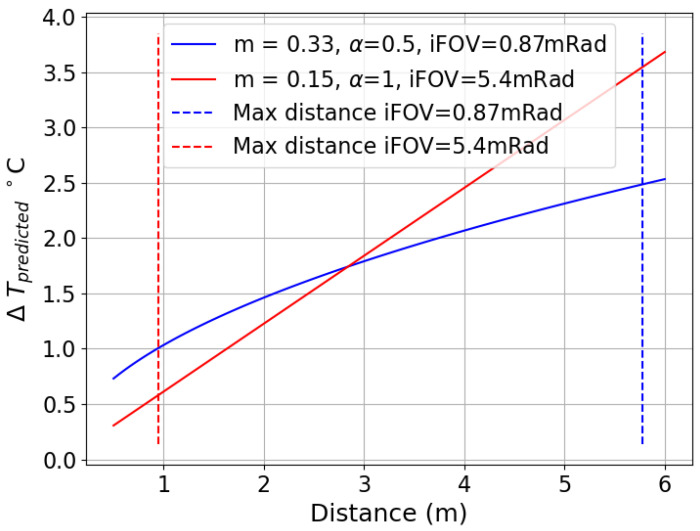
Effect of MSSE artifact on accuracy for human body thermometry of two systems as a function of distance to target, with vertical dotted lines indicating maximum distance threshold to enable a 5 mm spot size. Data to left of a vertical dotted line would meet the required spot size for the same color system plotted.

**Figure 11:**
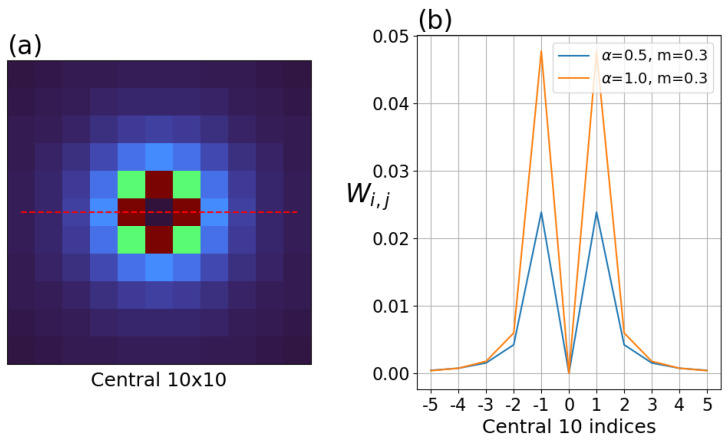
(**a**) Central 10 × 10 area of kernel generated with α = 0.5 with dotted line drawn to indicate profile selection. (**b**) Profiles indicated in (**a**) for kernels with α = 0.5 and 1.0.

**Figure 12:**
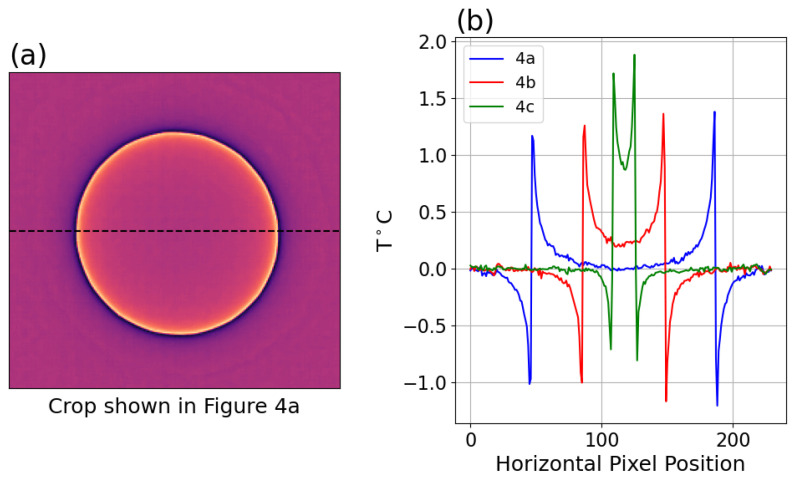
(**a**) Magnitude of correction (difference between corrected and uncorrected data) as image for $T_{T}$ = 50 °C setpoint, the dotted line showing pixel locations of profile data. (**b**) Profile of correction for large, medium, and small iris data as in Figure 4.

**Figure 13:**
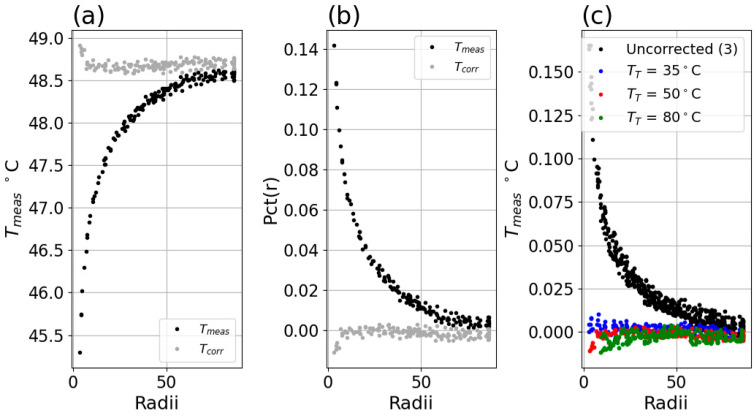
(**a**) Measured center temperature $T_{meas}$ versus radius for uncorrected data (black) and Equation (13) corrected data (gray) for Boson system with $T_{T}$ = 50 °C. (**b**) *Pct*(*r*) for uncorrected (black) and corrected (gray) data. (**c**) *Pct*(*r*) for uncorrected (black) and corrected (colored) data for $T_{T}$ = 35 (blue), 50 (red), and 80 (green) °C setpoints.

**Figure 14:**
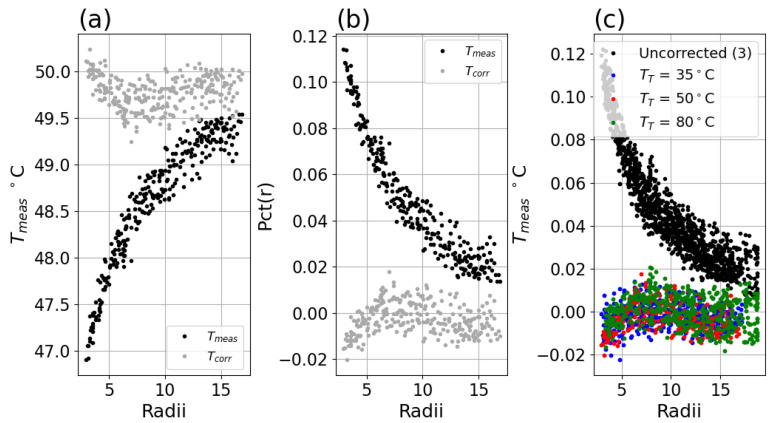
(**a**) Measured center temperature $T_{meas}$ versus radius for uncorrected data (black) and Equation (13) corrected data (gray) for Micro80 23° lens with $T_{T}$ = 50 °C. (**b**) *Pct*(*r*) for uncorrected (black) and corrected (gray) data. (**c**) *Pct*(*r*) for uncorrected (black) and corrected (colored) data for $T_{T}$ = 35 (blue), 50 (red), and 80 (green) °C setpoints.

**Figure 15:**
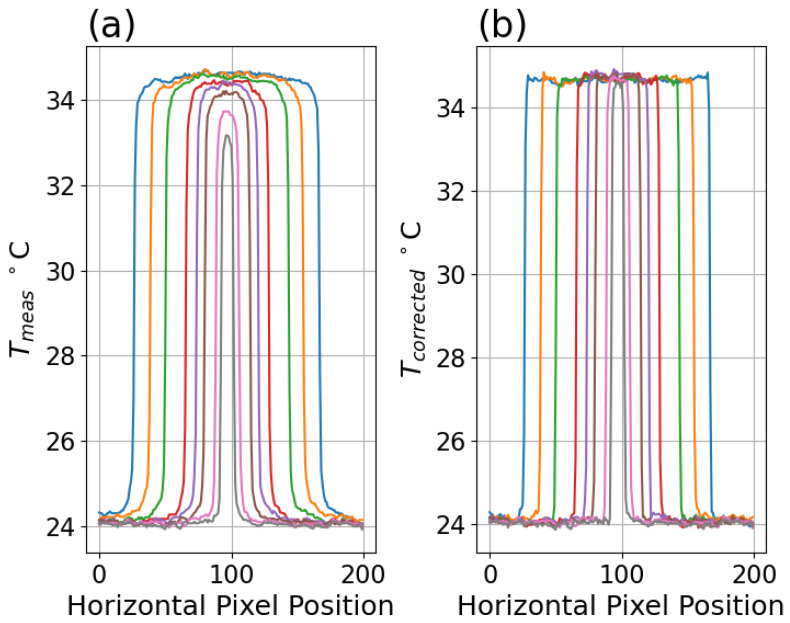
(**a**) Profiles of temperatures across midline of disks at various radii for uncorrected Boson with $T_{T}$ = 35 °C. (**b**) Profiles of same after correction applied.

**Figure 16:**
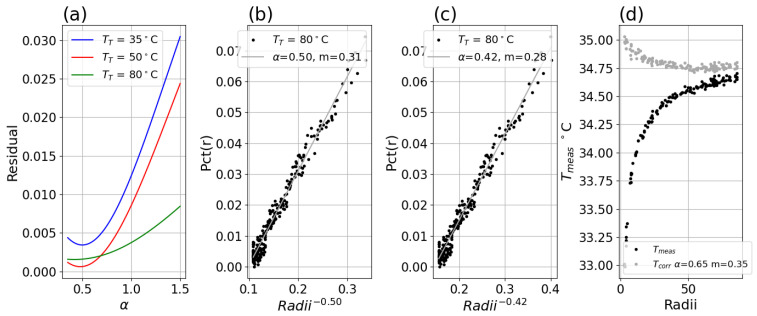
(**a**) Residual as a function of α for three setpoints with minimum of 0.50, 0.48 and 0.42 respectively; (**b**) *Pct*(*r*) for $T_{T}$ = 80 °C and α = 0.50 fit; (**c**) *Pct*(*r*) for $T_{T}$ = 80 °C and α = 0.42 fit; (**d**) measured center temperature $T_{meas}$ versus radius for uncorrected data (black) and Equation (13) corrected data (gray) for Boson system with $T_{T}$ = 35 °C for α = 0.65 fit.

**Figure 17:**
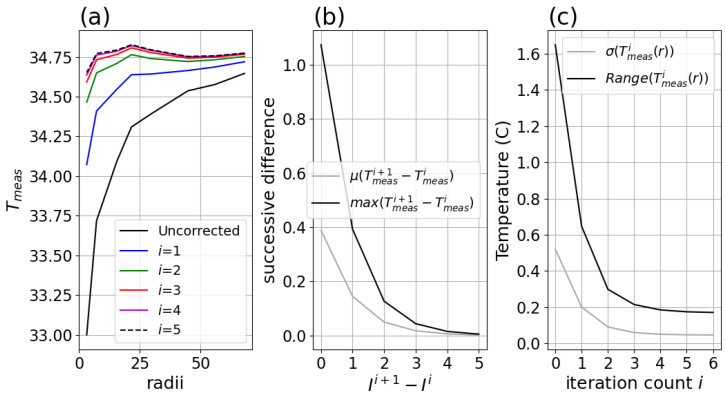
(**a**) $T_{meas}$ versus disk radii after successive iterations (up to 5) of Equation (12) applied to same images as used in profiles in Figure 15, (**b**) mean and maxima across radii of differences between successive iterations of correction applied prior to obtaining $T_{meas}$, (**c**) standard deviation and peak-to-peak range across radii versus iteration of correction applied before obtaining $T_{meas}$.

**Figure 18:**
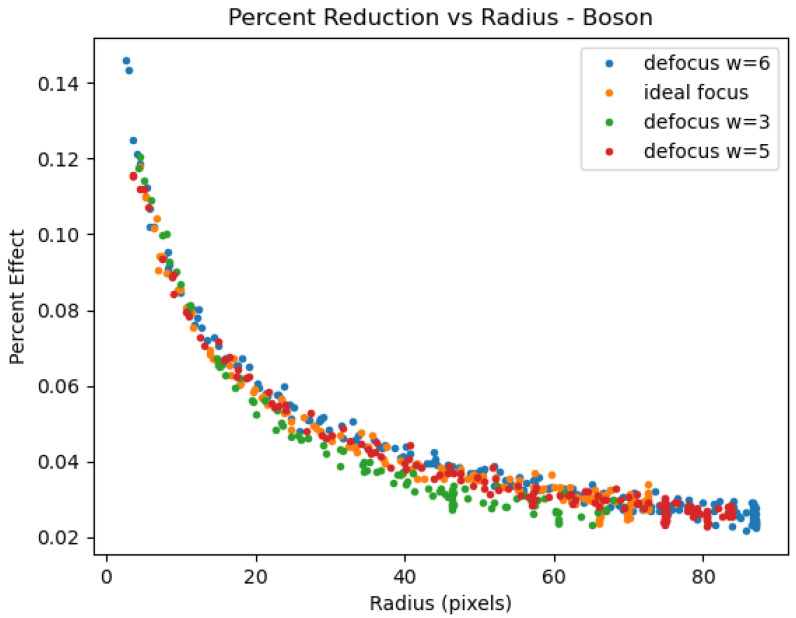
*Pct*(*r*) versus r for Boson system with $T_{T}$ = 35 °C data and different focus settings.

**Figure 19:**
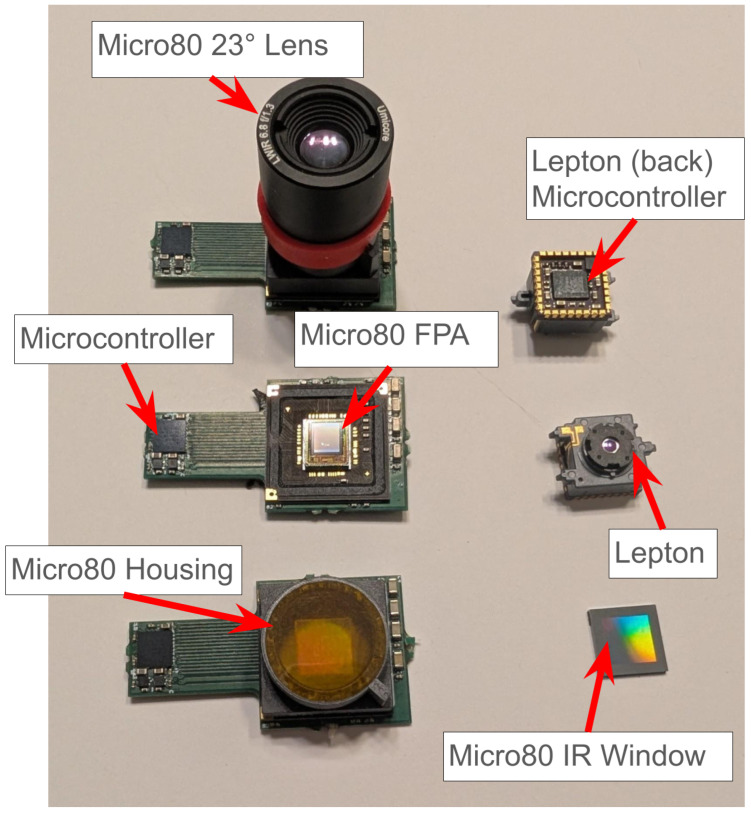
Lower-resolution systems used in study. Note the location of the microcontroller on the backside of Lepton, as optical housing for both are constructed of plastic (lower left shows optical housing for Micro80, Kapton tape over IR window and lens mount is present for handling during postprocessing). The 23 ° lens is mounted in the optical housing in the upper left.

**Table 1:** IR systems used in measurements.

Sensor/Pitch	Pixel Array	hFOV/iFOV	Lens	f-Number
Boson 12 μm	640 × 512	32/0.87 mRad	13.6 mm	f/1.0
Lepton 17 μm	80 × 60	50/11.64 mRad	1.46 mm	f/1.1
Micro80 34 μm	80 × 80	23/5.02 mRad	6.8 mm	f/1.3
Micro80 34 μm	80 × 80	39/8.51 mRad	4.0 mm	f/1.2
Micro80 34 μm	80 × 80	25/5.45 mRad	6.2 mm	f/1.1

**Table 2:** Correction results.

Sensor/hFOV	Setpoint	m	σ/$\sigma_{corr}$ °C	$R/R_{corr}$ °C
Boson 32 μm	35 °C	0.32	0.36/0.04	0.93/0.13
Boson 32 μm	50 °C	0.295	0.60/0.05	1.76/0.16
Boson 32 μm	80 °C	0.27	0.79/0.129	2.39/0.42
Lepton 50 μm	35 °C	0.29	0.57/0.23	1.88/0.74
Lepton 50 μm	50 °C	0.26	1.35/0.61	4.29/1.91
Lepton 50 μm	80 °C	0.235	3.74/2.09	12.34/7.00
Micro80 23 μm	35 °C	0.27	0.28/0.07	0.90/0.23
Micro80 23 μm	50 °C	0.28	0.66/0.18	2.11/0.54
Micro80 23 μm	80 °C	0.28	1.65/0.47	5.59/1.50
Micro80 39 μm	35 °C	0.165	0.14/0.05	0.46/0.17
Micro80 39 μm	50 °C	0.175	0.39/0.16	1.16/0.54
Micro80 39 μm	80 °C	0.18	0.97/0.28	3.16/0.87
Micro80 25 μm	35 °C	0.175	0.13/0.04	0.45/0.14
Micro80 25 μm	50 °C	0.155	0.29/0.13	1.00/0.39
Micro80 25 μm	80 °C	0.18	0.81/0.29	2.71/1.03

**Figure A1:**
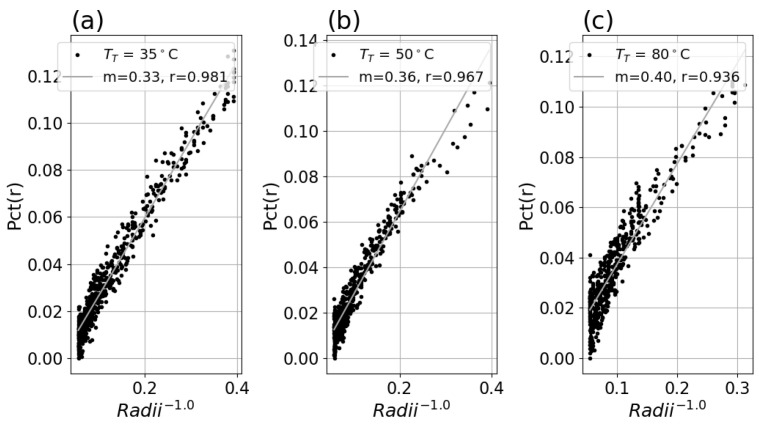
(**a**) Pct(r) versus $r^{-1}$ for 23° Micro80 system with $T_{T}$ = 35 °C setpoint, with fitted slope and linear correlation in the legend. (**b**) *Pct*(*r*) versus $r^{-1}$ for $T_{T}$ = 50 °C data. (**c**) *Pct*(*r*) versus $r^{-1}$ for $T_{T}$ = 80 °C data.

**Figure A2:**
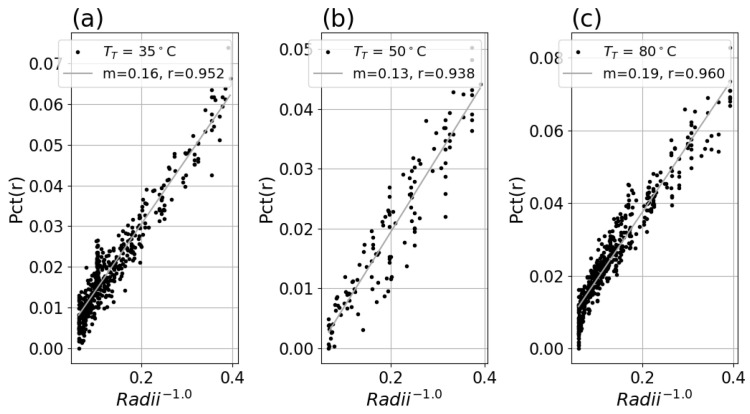
(**a**) Pct(r) versus $r^{-1}$ for 25° Micro80 system with $T_{T}$ = 35 °C setpoint, with fitted slope and linear correlation in the legend. (**b**) *Pct*(*r*) versus $r^{-1}$ for $T_{T}$ = 50 °C data. (**c**) *Pct*(*r*) versus $r^{-1}$ for $T_{T}$ = 80 °C data.

**Figure A3:**
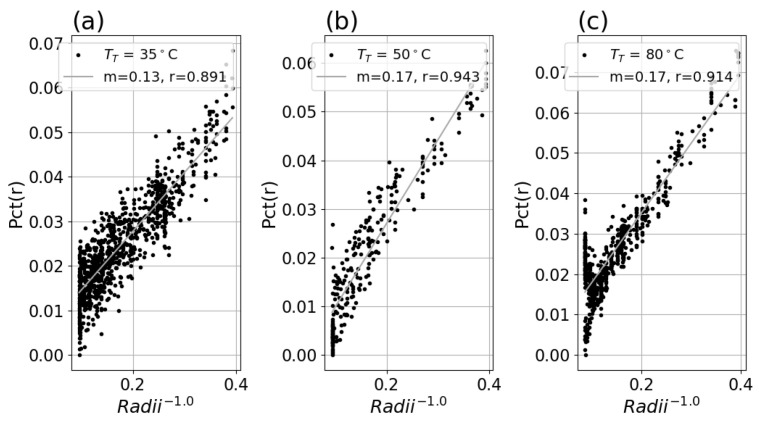
(**a**) Pct(r) versus $r^{-1}$ for 39° Micro80 system with $T_{T}$ = 35 °C setpoint, with fitted slope and linear correlation in the legend. (**b**) *Pct*(*r*) versus $r^{-1}$ for $T_{T}$ = 50 °C data. (**c**) *Pct*(*r*) versus $r^{-1}$ for $T_{T}$ = 80 °C data.

**Figure A4:**
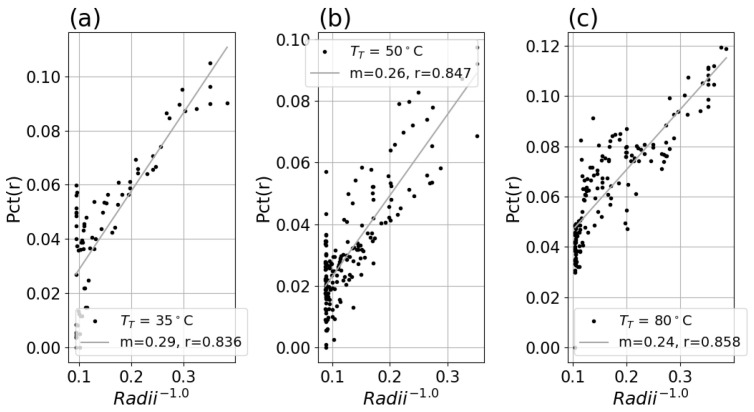
(**a**) Pct(r) versus $r^{-1}$ for Lepton system with $T_{T}$ = 35 °C setpoint, with fitted slope and linear correlation in the legend. (**b**) *Pct*(*r*) versus $r^{-1}$ for $T_{T}$ = 50 °C data. (**c**) *Pct*(*r*) versus $r^{-1}$ for $T_{T}$ = 80 °C data.

**Figure A5:**
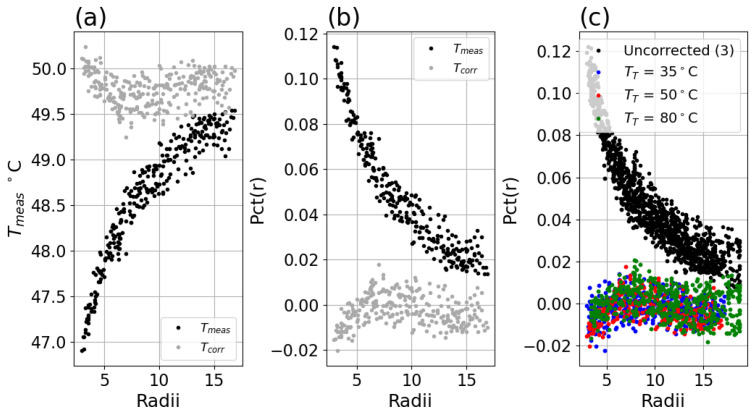
(**a**) Measured center temperature $T_{meas}$ versus radius for uncorrected data (black) and Equation (13) corrected data (gray) for Micro80 23° lens with $T_{T}$ = 50 °C. (**b**) *Pct*(*r*) for uncorrected (black) and corrected (gray) data. (**c**) *Pct*(*r*) for uncorrected (black) and corrected (gray) data for $T_{T}$ = 35 (blue), 50 (red), and 80 (green) °C setpoints.

**Figure A6:**
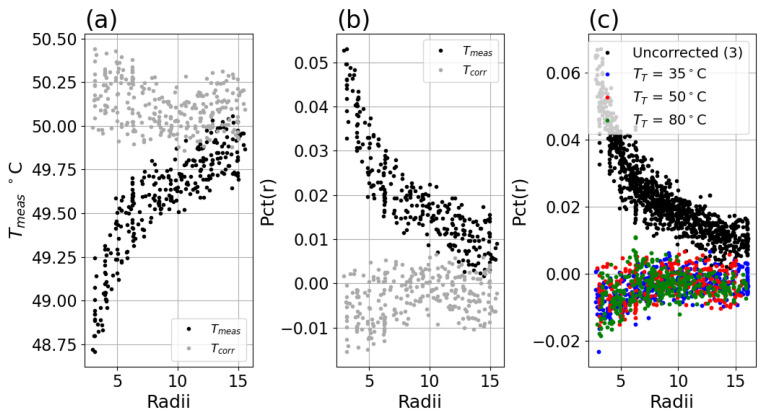
(**a**) Measured center temperature $T_{meas}$ versus radius for uncorrected data (black) and Equation (13) corrected data (gray) for Micro80 25° lens with $T_{T}$ = 50 °C. (**b**) *Pct*(*r*) for uncorrected (black) and corrected (gray) data. (**c**) *Pct*(*r*) for uncorrected (black) and corrected (gray) data for $T_{T}$ = 35 (blue), 50 (red), 80 (green) °C setpoints.

**Figure A7:**
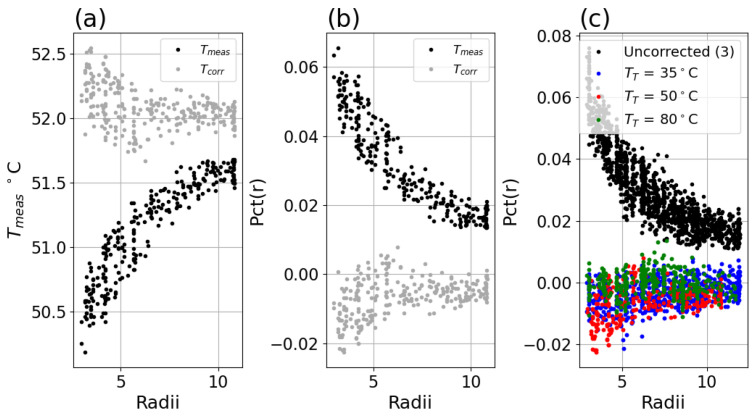
(**a**) Measured center temperature $T_{meas}$ versus radius for uncorrected data (black) and Equation (13) corrected data (gray) for Micro80 39° lens with $T_{T}$ = 50 °C. (**b**) *Pct*(*r*) for uncorrected (black) and corrected (gray) data. (**c**) *Pct*(*r*) for uncorrected (black) and corrected (gray) data for $T_{T}$ = 35 (blue), 50 (red), and 80 (green) °C setpoints.

**Figure A8:**
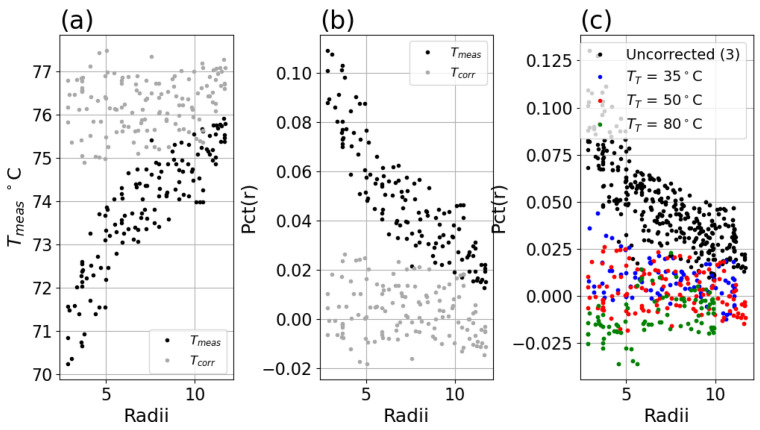
(**a**) Measured center temperature $T_{meas}$ versus radius for uncorrected data (black) and Equation (13) corrected data (gray) for Lepton system with $T_{T}$ = 50 °C. (**b**) *Pct*(*r*) for uncorrected (black) and corrected (gray) data. (**c**) *Pct*(*r*) for uncorrected (black) and corrected (gray) data for $T_{T}$ = 35 (blue), 50 (red), and 80 (green) °C setpoints.

## Data Availability

The datasets and analyses used in this study are available from a publicly available repository hosted at https://www.github.com/erikbeall/sse_paper (accessed on 27 March 2026).

## Abbreviations

The following abbreviations are used in this manuscript:

SSE	Size-of-source effect
SSSE	Single-element SSE
MSSE	Multi-element SSE
IR	Infrared
LWIR	Long-wavelength IR
MAD	Mean Absolute Difference
FOV	Field of view
iFOV	Instantaneous FOV
PSF	Point Spread Function
MTF	Modulation Transfer Function
FPA	Focal plane array
ADC	Analog to digital converter
NUC	Non-Uniformity Correction
ETRS	External Temperature Reference Source
$T_{BG}$	Background temperature
$T_{T}$	Target temperature
Pct	Percent reduction in temperature
FF	Full-field illumination
PF	Partial-field illumination

## Appendix A. Additional Plots

The main body of this article contained only the plots of selected systems and setpoints. The full set of *Pct*(*r*) fits for the low resolution systems listed in Table 1 are as follows:

The full set of correction result plots for the low resolution systems in Table 1 and Table 2 are as follows:
